# Higher chances of survival to hospital admission after out-of-hospital cardiac arrest in patients with previously diagnosed heart disease

**DOI:** 10.1136/openhrt-2021-001805

**Published:** 2021-12-21

**Authors:** Laura H van Dongen, Marieke T Blom, Sandra C M de Haas, Henk C P M van Weert, Petra J M Elders, Hanno L Tan, For the ESCAPE-NET Investigators

**Affiliations:** 1 Department of Cardiology, Heart Center, Amsterdam UMC Location AMC, Amsterdam, North Holland, The Netherlands; 2 Department of General Practice, Amsterdam Public Health, Amsterdam UMC Locatie AMC, Amsterdam, The Netherlands; 3 General Practice Medicine, Amsterdam UMC Locatie VUmc, Amsterdam, Noord-Holland, The Netherlands; 4 Netherlands Heart Institute, Utrecht, The Netherlands

**Keywords:** out-of-hospital cardiac arrest, ventricular fibrillation, epidemiology, heart disease, Survival

## Abstract

**Aim:**

This study aimed to determine whether patients suffering from out-of-hospital cardiac arrest (OHCA) with a pre-OHCA diagnosis of heart disease have higher survival chances than patients without such a diagnosis and to explore possible underlying mechanisms.

**Methods:**

A retrospective cohort study in 3760 OHCA patients from the Netherlands (2010–2016) was performed. Information from emergency medical services, treating hospitals, general practitioner, resuscitation ECGs and civil registry was used to assess medical histories and the presence of pre-OHCA diagnosis of heart disease. We used multivariable regression analysis to calculate associations with survival to hospital admission or discharge, immediate causes of OHCA (acute myocardial infarction (AMI) vs non-AMI) and initial recorded rhythm.

**Results:**

Overall, 48.1% of OHCA patients had pre-OHCA heart disease. These patients had higher odds to survive to hospital admission than patients without pre-OHCA heart disease (OR 1.25 (95%CI 1.05 to 1.47)), despite being older and more often having cardiovascular risk factors and some non-cardiac comorbidities. These patients also had higher odds of shockable initial rhythm (SIR) (OR 1.60 (1. 36 to 1.89)) and a lower odds of AMI as immediate cause of OHCA (OR 0.33 (0.25 to 0.42)). Their chances of survival to hospital discharge were not significantly larger (OR 1.16 (0.95 to 1.42)).

**Conclusion:**

Having pre-OHCA diagnosed heart disease is associated with better odds to survive to hospital admission, but not to hospital discharge. This is associated with higher odds of a SIR and in a subgroup with available diagnosis a lower proportion of AMI as immediate cause of OHCA.

**Key questions**

**What is already known about this subject?**

- Earlier recognition of individuals at risk of out-of-hospital cardiac arrest (OHCA) is advocated, but it is poorly studied how having a diagnosis of heart disease impacts on survival chances after OHCA. One previous study showed that patients with a diagnosis of ischaemic heart disease prior to OHCA had higher chances of survival. It is unknown whether higher survival chances apply to heart conditions in general and what mechanisms may underlie this association.

**What does this study add?**

- This study found that pre-OHCA diagnosed heart disease was associated with better odds to survive to hospital admission and that this is associated with higher odds of a shockable initial rhythm and lower proportion of acute myocardial infarction as immediate cause of OHCA, despite comparable resuscitation characteristics in patients with or without a pre-OHCA diagnosis of heart disease. However, the survival benefit did not persist to hospital discharge.

**How might this impact on clinical practice?**

- This study provides insight into potential mechanisms for survival and a lead for the development of novel preventative and/or treatment strategies.

## Introduction

Out-of-hospital cardiac arrest (OHCA) is a public health problem affecting 275 000 patients per year in Europe.^1^ Because survival rates after OHCA remain low (5%–22%), despite recent advancements in resuscitation care,^1^ identifying other factors that influence survival might benefit the development of management and preventative strategies.

Patient characteristics have been suggested to influence survival chances. As heart disease is a major risk factor for the occurrence of OHCA, it is conceivable that it may also influence the chances to survive OHCA. At present, one study found that having diagnosed ischaemic heart disease prior to OHCA was associated with higher chances of survival to hospital discharge.^2^ In contrast, another study found that prior coronary artery disease or congenital heart disease was associated with poorer survival.^3^ Due to these contradictory results, this study aimed to shed light on the association between a pre-OHCA diagnosis of heart disease and survival chances. Additionally, it is unknown whether this association is generalisable to being diagnosed with any heart disease and what mechanisms may underlie this association.

Survival after OHCA depends on many factors, in particular, the presence of a shockable initial rhythm (SIR), that is, ventricular fibrillation or ventricular tachycardia. Presence of SIR, in turn, is not only determined by situational factors (eg, presence of a witness and/or basic life support prior to emergency medical services (EMS) arrival, EMS response time) but also by patient characteristics (e.g., age, sex, comorbidities).^4–8^ Prior treatment by a cardiologist may change the likelihood of SIR and thereby survival chances by changing the distribution of underlying causes of OHCA. In particular, we focused on the occurrence of acute myocardial infarction (AMI), the most common immediate cause of OHCA.^9–12^

The aim of our study was to investigate whether having a diagnosis of heart disease prior to OHCA (pre-OHCA heart disease) is associated with a higher chance to survive OHCA. We also studied possible mechanisms that may underlie an association, focusing on possible changes in the proportion of SIR and the proportion of underlying AMI.

## Methods

### Study population and setting

A retrospective cohort study was performed using data from the AmsteRdam REsuscitation STudies (ARREST) registry, an ongoing prospective registry of all EMS-attended OHCA cases in one contiguous region of the Netherlands (North Holland province, urban and rural areas, 2.4 million inhabitants) in the period 1 January 2010 to 31 December 2016. A detailed description of this cohort is provided elsewhere.^13^ Patients were excluded when their OHCA had a non-cardiac cause (eg, trauma, asphyxiation, drug overdose), they were under 18 years, lived abroad, had irretrievable medical history or missing data for confounders or outcome (study population 1) or did not survive to hospital diagnosis (study population 2).

### Data collection

The medical history prior to OHCA was retrieved from the general practitioner (GP) and contained the patients’ episode list (coded using the International Classification of Primary Care) and/or letters from medical specialists, along with a questionnaire filled out by the GP. This questionnaire contained a checklist of several comorbidities classified as yes versus no, including heart disease (AMI, atrial arrhythmias including atrial fibrillation/flutter, cardiomyopathy, congenital heart disease, ventricular arrhythmias, valvular heart disease, heart failure, angina pectoris) and cardiovascular risk profile (hypercholesterolemia, obesity, hypertension, diabetes mellitus type 1 or type 2, stroke). When the GP stated that a patient had a certain condition, either in the checklist or episode list, this patient was defined as having this condition prior to OHCA. Moreover, other pre-existing conditions were collected from the questionnaire, including depression, chronic obstructive pulmonary disease, cancer, renal dysfunction and liver dysfunction (yes/no). Medical history was designated as irretrievable when a GP did not want to cooperate or when a patients’ GP could not be ascertained.

The resuscitation parameters were collected according to the Utstein criteria^14^ and included: bystander or ambulance witnessed OHCA (yes/no), OHCA location (home vs public), bystander cardiopulmonary resuscitation (CPR, yes/no), connection of an automated external defibrillator (AED, yes/no) and time to defibrillator connection (time from emergency call to time of connection of AED or manual defibrillator, whichever came first).

### Exposure

Patients were classified as having pre-OHCA heart disease when their medical history showed the presence of one or more of the following conditions at any time before OHCA: AMI, atrial arrhythmia, cardiomyopathy, congenital heart disease, ventricular arrhythmia, valvular heart disease, heart failure or angina pectoris.

### Outcomes

The primary outcomes were survival to hospital admission and survival to hospital discharge and these were retrieved from hospital records. Secondary outcomes were SIR and AMI as immediate cause of OHCA. Initial rhythm was determined from the ECGs of the AED or manual defibrillator, whichever was connected to the patient first, and defined as SIR or non-SIR (pulseless electrical activity, asystole and bradycardia). The immediate cause of OHCA was established in the subset of patients who survived to hospital admission and was derived from hospital records (including letters, ECGs, cardiac enzymes and treatments), because it could not be reliably established for patients who died before hospital admission. The cause of OHCA was categorised as either AMI (ST-elevation AMI, non-ST-elevation AMI or AMI not specified) or non-AMI. These categories were chosen because AMI is the most prevalent cause of OHCA (56%), while the other causes were very low in number. We abided by the diagnosis made by the treating physicians as reported in the hospital letters, as we recognised that it is often difficult to distinguish between AMI as being the cause of OHCA and myocardial damage (evidenced by, for example, cardiac enzymes, ECG, echocardiography) occurring as a consequence of the cardiac arrest. Therefore, we felt that the hospital diagnosis is more likely to be correct than a diagnosis made by us based on less available information. Survival to hospital diagnosis was defined as survival to hospital admission with an established cause of OHCA, thereby excluding those patients who died before a cause could be established and patients for whom no cause could be retrieved due to missing hospital correspondence.

### Statistical analysis

First, we determined the proportion of patients with pre-OHCA heart disease, stratified by sex and initial rhythm. Patient and comorbidity characteristics were calculated comparing patients with pre-OHCA heart disease to patients without pre-OHCA heart disease. Continuous variables are presented as mean±SD or median (IQR) when appropriate and categorical variables as n (%). Statistical comparisons were made using independent sample t-tests, Kolmogorov-Smirnov test or χ^2^ statistics, where appropriate.

Second, we assessed the association between having pre-OHCA heart disease and (1) survival to hospital admission, (2) survival to hospital discharge, (3) presence of SIR (in study population 1) and (4) AMI as cause of OHCA (in study population 2), using multivariable logistic regression analyses. Five different models were used by subsequently adding various parameters. Model 1 adjusted for sex and age. In model 2, resuscitation parameters (OHCA location, bystander or ambulance witnessed OHCA, bystander CPR, connection of AED, time between emergency call and first recording of rhythm) were added. In model 3, initial rhythm (SIR vs non-SIR) was added to investigate effect modification by initial rhythm. Additionally, a fourth model was made, adjusting for model 3 and adding significant cardiovascular disease (CVD) risk factors (obesity, hypertension, hypercholesterolemia, stroke, type 2 diabetes) to the model and a fifth model adjusted for significant non-cardiac comorbidities. Models 1 through 5 were used to assess the association between pre-OHCA heart disease and survival to hospital admission or discharge and cause of OHCA. Models 1, 2, 4 and 5 (minus initial rhythm) were used to study the association between pre-OHCA heart disease and initial rhythm.

Stratification by initial rhythm was performed for the analyses regarding survival to hospital admission and discharge (models 1 and 2), as overall patients with SIR have better chances of survival. Patients were excluded from the stratified analyses if EMS witnessed the OHCA, as the presence of EMS witness was very differently distributed between SIR and non-SIR (1.4% vs 12.9%, p value<0.001).

All statistical analyses were performed using SPSS V.26.0 (SPSS). Two-sided p values<0.05 indicated statistical significance.

## Results

During the study period, a total of 3760 OHCA patients met the inclusion criteria (study population 1, online supplemental eFigure 1). In this total study population, the proportion of patients with pre-OHCA heart disease was 48.1% (men 48.3%, women 47.5%, p value=0.66). Patients with pre-OHCA heart disease were older and more often had a high CVD risk profile and some non-cardiac comorbidities (depression, chronic obstructive pulmonary disease, cancer, renal dysfunction) than patients without pre-OHCA heart disease (table 1). In addition, they had more often SIR, although there were no between-group differences in key determinants of the presence of SIR (male sex, time to defibrillator connection, location of OHCA and the proportions of bystander witnessed OHCA, bystander CPR provision and AED connection).

10.1136/openhrt-2021-001805.supp2 Supplementary data

**Table 1:** Characteristics of study population

	Without pre-OHCA diagnosed heart disease	With pre-OHCA diagnosed heart disease	P value
	n=1952	n=1808	
Age in years, mean±SD	64.7±14.0	71.4±12.6	<0.001
Male sex	1379 (70.6)	1289 (71.3)	0.66
Cardiovascular risk profile			
Obesity	311 (15.9)	406 (22.5)	<0.001
Hypertension	824 (42.2)	1073 (59.3)	<0.001
Hypercholesterolemia	498 (25.5)	690 (38.2)	<0.001
Stroke/transient ischaemic attack	168 (8.6)	319 (17.6)	<0.001
Type 2 diabetes mellitus	324 (16.6)	520 (28.8)	<0.001
Non-cardiac comorbidities			
Depression	138 (7.1)	220 (12.2)	<0.001
Chronic obstructive pulmonary disease	283 (14.5)	384 (21.2)	<0.001
Cancer	312 (16.0)	378 (20.9)	<0.001
Rheumatic disease	79 (4.0)	95 (5.3)	0.08
Renal dysfunction	136 (7.0)	400 (22.1)	<0.001
Liver dysfunction	73 (3.7)	92 (5.1)	0.13
Resuscitation parameters			
Witnessed arrest			0.06
By bystander	1250 (64.0)	1223 (67.6)	
By ambulance	154 (7.9)	135 (7.5)	
Bystander CPR provided	1482 (75.9)	1339 (74.1)	0.19
OHCA at home location	1404 (71.9)	1351 (74.7)	0.053
AED connected	1101 (56.4)	987 (54.6)	0.26
Shockable initial rhythm present	849 (43.5)	854 (47.2)	0.021
Time to defibrillator connection in min*, median (IQR)	8.5 (6.5–11.0)	8.5 (6.6–10.9)	0.67

Results are presented as n (%) unless indicated otherwise.

*Time between emergency call and connection of AED or manual defibrillator.

AED, automated external defibrillator; CPR, cardiopulmonary resuscitation; OHCA, out-of-hospital cardiac arrest.

**Figure 1:**
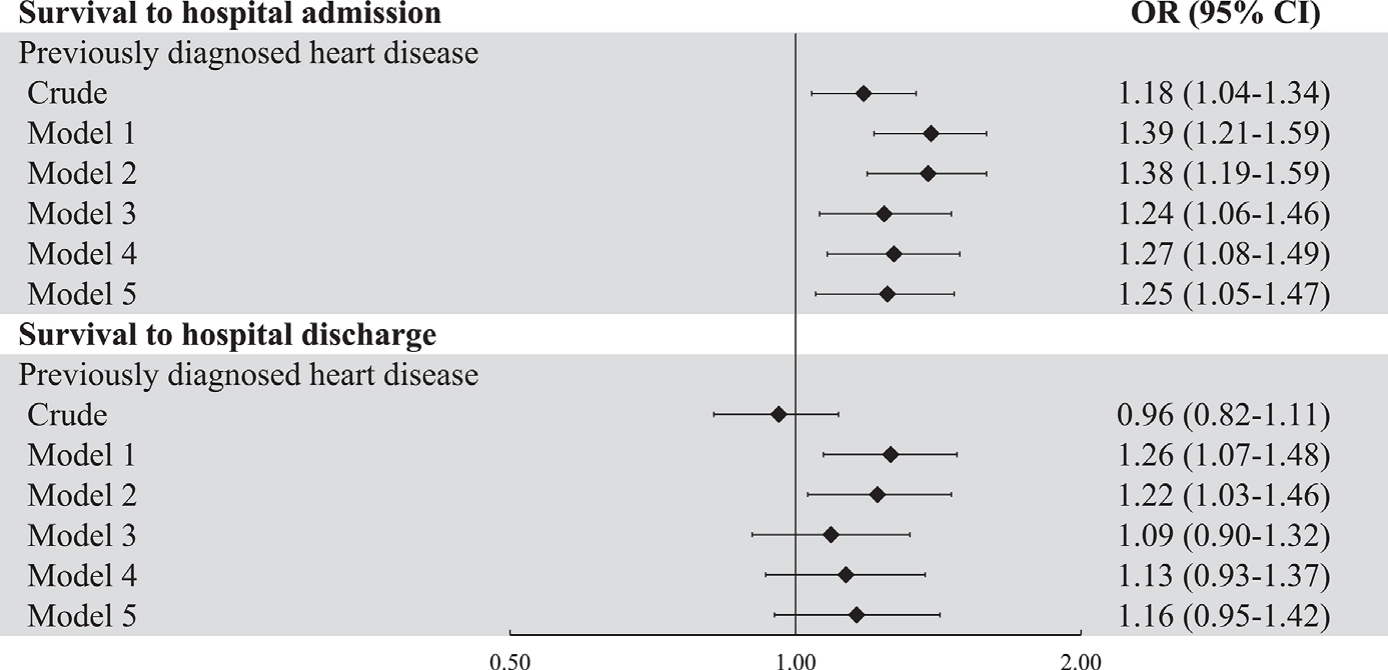
Forest plot of the associations between having pre-OHCA diagnosed heart disease and survival to hospital admission and survival to hospital discharge. Model 1 adjusted for age and sex. Model 2 adjusted for model 1 plus resuscitation parameters (use of automated external defibrillator, presence of bystander or ambulance witness, provision of bystander CPR, OHCA location and time to defibrillator connection) and model 3 for model 2 plus initial rhythm. Model 4 adjusted for model 3 plus significant cardiovascular risk profile (obesity, hypertension, hypercholesterolemia, stroke, type 2 diabetes) and model 5 additionally adjusted for significant non-cardiac comorbidities (depression, COPD, cancer and renal dysfunction). COPD, chronic obstructive pulmonary disease; CPR, cardiopulmonary resuscitation; OHCA, out-of-hospital cardiac arrest.

Among patients with pre-OHCA heart disease, 41.9% survived to hospital admission and 23.0% to hospital discharge, as compared with 38.0% and 23.8%, respectively, of patients without pre-OHCA heart disease (p values 0.014 and 0.53). Having pre-OHCA heart disease was associated with increased odds of survival to hospital admission after adjustment for resuscitation factors (model 2) with an OR of 1.38 (95% CI: 1.19 to 1.59, figure 1). However, after additional adjustment for initial rhythm (model 3), this association became weaker (OR 1.24, 95% CI: 1.06 to 1.46). In contrast, adding the comorbidities (CVD risk factors and non-cardiac comorbidities) that differed between both groups to the model did not change the association (OR 1.25 (1.05 to 1.47)). Pre-OHCA diagnosis of heart disease was not significantly associated with survival to hospital discharge in the fully adjusted model (OR 1.16 (0.95 to 1.42)). The addition of CVD risk factors and non-cardiac comorbidities altered the OR from 1.09 (0.90 to 1.32) to 1.16 (0.95 to 1.42).

**Figure 2:**
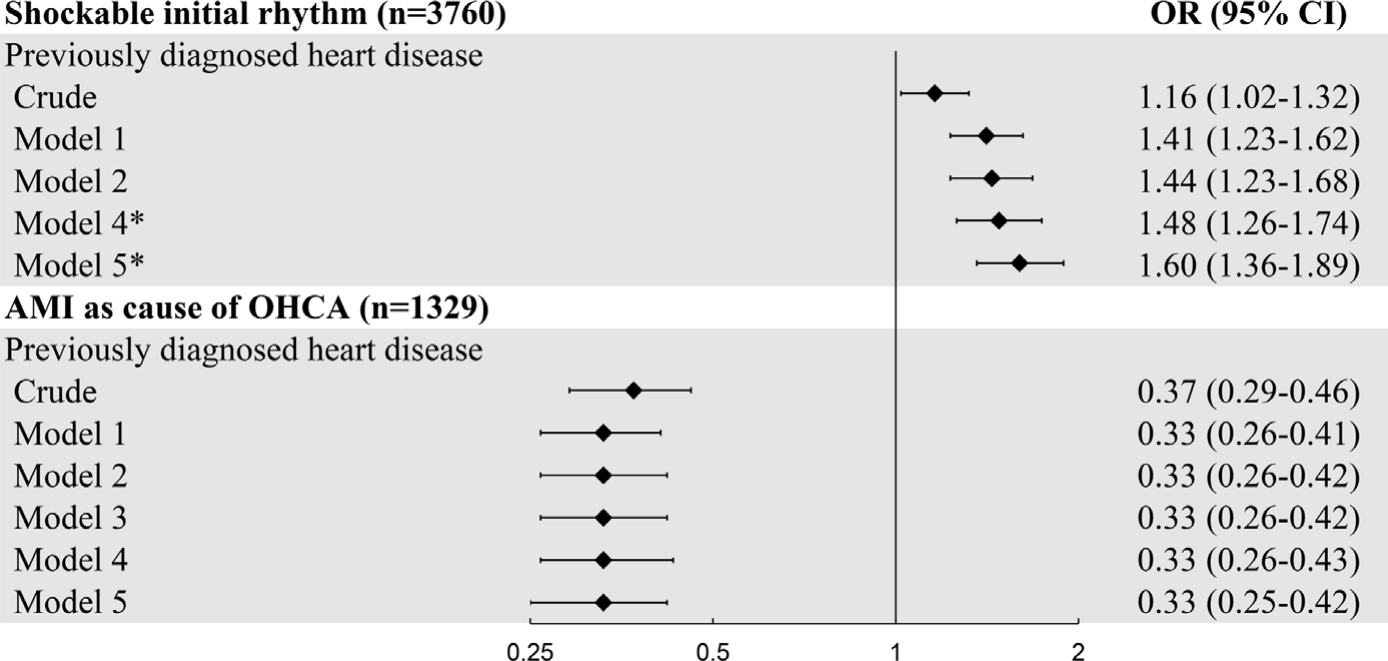
Forest plot of the association between having pre-OHCA diagnosed heart disease and shockable initial rhythm (SIR) and acute myocardial infarction (AMI) as immediate cause of OHCA. Model 1 adjusted for age and sex. Model 2 adjusted for model 1 plus resuscitation parameters (use of automated external defibrillator, presence of bystander or ambulance witness, provision of bystander CPR, OHCA location and time to defibrillator connection) and model 3 for model 2 plus initial rhythm. Model 4 adjusted for model 3 plus significant comorbidities (obesity, hypertension, hypercholesterolemia, stroke, type 2 diabetes) and model 5 additionally adjusted for significant non-cardiac comorbidities (depression, COPD, cancer and renal dysfunction). *Models 4 and 5: in the analysis on SIR the models do not adjust for initial rhythm as confounder. COPD, chronic obstructive pulmonary disease; CPR, cardiopulmonary resuscitation; OHCA, out-of-hospital cardiac arrest.

Furthermore, the association between initial rhythm and survival was analysed in patients with pre-OHCA heart disease and those without in more detail in patients from study population 1 whose OHCA onset was not EMS-witnessed (n=3471). Among the 1680 patients with SIR, having pre-OHCA heart disease was not significantly associated with survival to hospital admission (OR 1.12 (0.90 to 1.39)) or discharge (OR 1.08 (0.87 to 1.34)) in model 2 (online supplemental table 1). However, in our unadjusted analysis, we did observe a difference in the proportion of survival to hospital discharge between patients with or without pre-OHCA heart disease (41.8% vs 47.0%, p value 0.032). Among the 1791 patients without SIR, having pre-OHCA heart disease was associated with higher odds to survive to hospital admission (17.9% vs 11.6%, OR 1.76 (1.34 to 2.32) and survival to hospital discharge (0.8% vs 2.9%, OR 4.51 (1.95 to 10.40)); however, the number of patients without SIR who survived was small (n=149, online supplemental table 1). Moreover, the proportion of OHCA victims with SIR was larger in the group of patients with pre-OHCA heart disease (47.2%) than in the group of patients without (43.5%, p value 0.02). In all multivariable models, having pre-OHCA heart disease was positively associated with SIR, with an OR of 1.60 (95% CI: 1.36 to 1.89) in the fully adjusted model (figure 2), even though key resuscitation parameters that determine the odds of SIR were not different between both groups (table 1). The addition of CVD risk factors and non-cardiac comorbidities to the model increased the estimated OR by 14% (model 4: OR 1.44, model 5: OR 1.60).

10.1136/openhrt-2021-001805.supp1 Supplementary data

In our final analysis, we compared the proportions of patients whose immediate cause of OHCA was AMI between the groups of patients with or without pre-OHCA heart disease. We studied this in the subgroup of 1329 patients who survived to hospital diagnosis to ensure that presence/absence of AMI was correctly ascertained (study population 2, online supplemental eFigure 1). In this subgroup, the differences between the group with pre-OHCA heart disease and the group without were consistent with those observed in the total study population (study population 1). Thus, patients with pre-OHCA heart disease had higher age and higher CVD risk profile, but the resuscitation parameters were similar with the exception of a higher proportion of bystander witnessed OHCA (online supplemental table 2). Among patients with pre-OHCA heart disease, the proportion of patients with AMI as immediate cause of OHCA was lower (43.7%) than in the group without pre-OHCA heart disease (68.0%, p value<0.001). Accordingly, pre-OHCA diagnosed heart disease was associated with a lower proportion of AMI with an OR of 0.33 (0.25 to 0.42) in the fully adjusted model (figure 2). The ORs only marginally changed between models 1 and 5 (figure 2).

## Discussion

The main findings of this study are: (1) individuals with a pre-OHCA diagnosis of heart disease have higher chance to survive to hospital admission after OHCA than individuals without such a diagnosis, but not to hospital discharge; (2) this higher survival chance is partly determined by higher likelihood of SIR and (3) in the subgroup of patients who survived to hospital diagnosis, AMI is less often the immediate cause of OHCA in patients with a pre-OHCA diagnosis of heart disease than in patients without such a diagnosis.

Our finding that individuals who suffered OHCA have higher chances to survive to hospital admission if they had a pre-OHCA diagnosis of heart disease may be counterintuitive given that many factors disfavour their chances of survival: higher age, higher CVD risk profile and more non-cardiac comorbidities. Still, this finding has clinical relevance, as it provides a first indication that early recognition of individuals with elevated OHCA risk and subsequent earlier cardiologic treatment may impact survival after OHCA as well. Whether earlier cardiologic treatment affects OHCA risk needs confirmation in a (longitudinal) cohort study including both patients with and without an OHCA. Nevertheless, the present study shows that potential survival benefits may apply to having a pre-OHCA diagnosis of any cardiac condition and are not limited to ischaemic heart disease on which prevention programmes are heavily focused at present. Importantly, previous studies have indicated that earlier recognition may be feasible as many OHCA victims have pre-OHCA symptoms^15–18^ and contact the healthcare system shortly before OHCA,^19–21^ although their presenting signs and symptoms may be non-specific at that time. Moreover, one study indicated that a timely response to patients with symptoms (such as chest pain, dyspnoea or syncope) resulted in better survival.^15^ Still, most symptoms were not recognised as needing immediate action and were thus ignored. This suggests that efforts at better symptom recognition are warranted, which may result in better prediction tools for care providers.

We next investigated the possible mechanism underlying the better survival rates to hospital admission in patients with a pre-OHCA diagnosis of heart disease. We found that this improved outcome was associated with a higher proportion of SIR, while this survival gain was reduced when we corrected for initial rhythm. Similarly, in our stratified analyses in patients with SIR, no statistically significant difference in survival was observed between patients with pre-OHCA heart disease and those without. Together, these findings point to the important role of a higher proportion of SIR. This higher proportion in patients with pre-OHCA heart disease was not related to more favourable resuscitation parameters, as time to defibrillator connection, presence of witness, provision of bystander CPR or of AED connection was comparable between patients with or without pre-OHCA heart disease. Thus, it was more likely to stem from different patient factors between both groups. In our final analysis, we therefore studied whether the proportion of AMI as immediate cause of OHCA was lower in patients with pre-OHCA heart disease, because AMI is the most prevalent immediate cause of OHCA, while ischaemic heart disease constitutes a major category of heart disease, and the treatment of a large proportion of patients with pre-OHCA heart disease therefore focuses on ischaemic heart disease and the prevention of AMI. In accordance with these considerations, we found that AMI was less often the immediate cause of OHCA among patients with pre-OHCA heart disease.

Despite the higher chances of survival to hospital admission, the chances of survival to hospital discharge were comparable between patients with or without pre-OHCA heart disease. Although we did not study the underlying causes of the higher in-hospital mortality in patients with pre-OHCA heart disease, it is readily conceivable that it stems from the more unfavourable patient factors of these patients (higher age, higher CVD risk profile, more non-cardiac comorbidities).^17 20^ It may also relate to the type of pre-OHCA heart disease, as our exposure is very heterogeneous. However, a prior study that was limited to patients with a pre-OHCA diagnosis of ischaemic heart disease reported higher odds for survival to discharge in these patients.^2^ Also, other investigators reported that OHCA patients without AMI who survived to hospital admission had higher mortality rates than OHCA patients with AMI,^22^ which may be related to postcardiac arrest shock.^23^ Nevertheless, another study that was limited to patients with ischaemic heart disease and congenital heart disease reported a lower survival chance to hospital discharge in these patients.^3^ Increased prehospital survival rates may provide more treatment opportunities, but it is a matter of debate whether having a higher prehospital survival chance is positive for the OHCA victim and/or relatives if the overall survival chance remains unchanged. Despite all efforts already undertaken, more research is required to investigate why the immediate survival advantage does not translate into longer term survival. This may eventually result in improved measures in the prehospital^24 25^ or in-hospital^26^ phase to increase overall survival chances.

### Strengths and limitations

Our study has several strengths. First, it used data from the ARREST registry which was specifically designed to study determinants and outcomes of OHCA. This ensured an accurate OHCA diagnosis, including initial rhythm analysis from ECGs of AED and/or manual defibrillator. Additionally, we collected not only the medical history of patients who were transported to the hospital alive but also of those who died on the scene.

Our study also has some limitations. First, we only had data on the immediate cause of OHCA of patients who survived to hospital diagnosis, while autopsy results were rare (autopsy after OHCA is not mandatory and rarely conducted in the Netherlands^27^); this may have resulted in selection bias. Second, only patients with EMS who attended OHCA were included. This resulted in exclusion of individuals who were clearly already deceased when they were found and for whom EMS was not alerted. Also, in patients with implantable cardioverter defibrillators (ICDs) in whom OHCA was terminated by the ICD alone, EMS was not involved. Thus, only few patients with ICDs were included and our calculated survival benefit of the group of patients with pre-OHCA heart disease was likely an underestimation of their true survival benefit. Third, presence of a non-SIR at EMS contact does not mean that SIR was not present prior to connection of an AED or manual defibrillator. This could have led to misclassification. Fourth, we had only few non-SIR survivors in our stratified analyses, limiting the statistical power. Additionally, the questionnaire used to retrieve medical history from the patients GP may not precisely reflect the complete comorbidity status of the patient. Also, the classification of prior heart disease is very heterogeneous and the individual types of heart diseases included may have a stronger or weaker association with survival when assessed separately. Lastly, residual confounding may still exist despite our attempts to control for possible confounding factors and the nature of this study cannot infer causality.

## Conclusion

Patients with a pre-OHCA diagnosis of heart disease have better survival odds to hospital admission than patients in whom OHCA was the first manifestation of heart disease. This study shows that the survival gain to hospital admission applies to any cardiac condition and is not limited to ischaemic heart disease, on which prevention programmes are heavily focused at present. This survival gain is associated with a higher proportion of SIR, and in the subgroup of patients who survived to hospital diagnosis, it is associated with a lower proportion of AMI as immediate cause of OHCA. These findings lend support to the notion that earlier recognition and timely treatment of individuals at risk of OHCA might pay off and that we must work towards a greater ability to do so.

## Data Availability

The data underlying this article are available in the article and in its online supplementary material. The data cannot be shared publicly for privacy of individuals that participated in the study as data cannot be provided completely anonymous according to the Medical Ethics Committee and the Data Protection Officer of our institution.
