# Correction: *Higher chances of survival to hospital admission after out-of-hospital cardiac arrest in patients with previously diagnosed heart disease*

**DOI:** 10.1136/openhrt-2021-001805corr1

**Published:** 2022-01-25

van Dongen LH, Blom MT, de Haas SCM, ESCAPE-NET Investigators, *et al*. Higher chances of survival to hospital admission after out-of-hospital cardiac arrest in patients with previously diagnosed heart disease. *Open Heart* 2021;**8:**e001805.

Middle initials have been added to the following authors: Laura H van Dongen, Petra J M Elders, Hanno L Tan.

