# Supplementary material for: Higher chances of survival to hospital admission after out-of-hospital cardiac arrest in patients with previously diagnosed heart disease
Source: Open Heart. 2021 Dec 21;8(2):e001805. doi: 10.1136/openhrt-2021-001805 (PMC8693164; doi:10.1136/openhrt-2021-001805)
Supplement: Supplementary data [file openhrt-2021-001805supp001.pdf]

Supplemental Table 1. Multivariate logistic regression analyses of the association between having previously diagnosed heart disease and survival to hospital admission and discharge stratified by initial rhythm.

	**Survival to hospital admission**			
	SIR n=1680		Non-SIR n=1791	
	Survived	Died	Survived	Died
Without previously diagnosed heart disease, n (%)	550 (65.6)	288 (34.4)	111 (11.6)	849 (88.4)
With previously diagnosed heart disease, n (%)	550 (65.3)	292 (34.7)	149 (17.9)	682 (82.1)
P-value	0.89		<0.001	
Models, OR (95% CI)				
Crude	0.99 (0.81-1.21)		1.67 (1.28-2.18)	
Model 1	1.12 (0.90-1.38)		1.75 (1.33-2.31)	
Model 2	1.12 (0.90-1.39)		1.76 (1.34-2.32)	

	**Survival to hospital discharge**			
	SIR n=1680		Non-SIR n=1791	
	Survived	Died	Survived	Died
Without previously diagnosed heart disease, n (%)	394 (47.0)	444 (53.0)	8 (0.8)	952 (99.2)
With previously diagnosed heart disease, n (%)	352 (41.8)	490 (58.2)	24 (2.9)	807 (97.1)
P-value	0.032		0.001	
Odds ratio (95% CI)				
Crude	0.81 (0.67-0.98)		3.54 (1.58-7.92)	
Model 1	1.08 (0.88-1.33)		4.93 (2.13-11.37)	
Model 2	1.08 (0.87-1.34)		4.51 (1.95-10.40)	

Model 1 adjusted for age and sex. Model 2 adjusted for model 1 plus resuscitation parameters (use of automated external defibrillator, presence of bystander or ambulance witness, provision of bystander cardiopulmonary resuscitation, OHCA location, and time to defibrillator connection).

CI=confidence interval. OHCA=out-of-hospital cardiac arrest. OR=odds ratio. SIR=shockable initial rhythm.

Supplemental Table 2. Baseline characteristics of 1329 patients surviving to hospital diagnosis.

	Without previously diagnosed heart disease n=674	With previously diagnosed heart disease n=655	P-value
Age in years, mean ± SD	61.3 ± 12.7	68.0 ± 12.7	<0.001
Male sex	527 (78.2)	508 (77.6)	0.78
Cardiovascular disease risk profile			
Obesity	95 (14.1)	128 (19.5)	0.008
Hypertension	268 (39.8)	360 (55.0)	<0.001
Hypercholesterolemia	180 (26.7)	280 (42.7)	<0.001
Stroke/transient ischemic attack	33 (4.9)	82 (12.5)	<0.001
Type 2 diabetes	89 (13.2)	152 (23.2)	<0.001
Non-cardiac comorbidities			
Depression	43 (6.4)	75 (11.5)	0.001
Chronic obstructive pulmonary disease	85 (12.6)	105 (16.0)	0.075
Cancer	76 (11.3)	107 (16.3)	0.007
Rheumatic disease	24 (3.6)	34 (5.2)	0.15
Renal dysfunction	33 (4.9)	108 (16.5)	<0.001
Liver dysfunction	25 (3.7)	27 (4.1)	0.70
Resuscitation parameters			
Witnessed arrest			0.02
By bystander	516 (76.6)	542 (82.7)	
By ambulance	77 (11.4)	54 (8.2)	
Bystander CPR provided	512 (76.0)	513 (78.3)	0.31
OHCA at home location	370 (54.9)	380 (58.0)	0.25
AED connected	389 (57.7)	369 (56.3)	0.61
Shockable initial rhythm present	538 (79.8)	521 (79.5)	0.90
Time to defibrillator connection*, min, median (IQR)	7.8 (5.5-10.5)	7.8 (5.9-10.3)	0.25

Results are presented as n (%) unless indicated otherwise.

AED=automated external defibrillator. CPR=cardiopulmonary resuscitation. IQR=interquartile range. OHCA=out-of-hospital cardiac arrest. SD=standard deviation.

* Time between emergency call and connection of an AED or manual defibrillator.
